# An Evidence-Based Approach to the Management of Primary Ovarian Insufficiency in Adolescents and Young Women

**DOI:** 10.3390/life15091366

**Published:** 2025-08-28

**Authors:** Hanadi Bakhsh

**Affiliations:** 1 Obstetrics and Gynecology Department, College of Medicine, Princess Nourah bint Abdulrahman University, Riyadh 11564, Saudi Arabia; drobgyn2005@gmail.com; 2 Department of Obstetrics and Gynecology, King Abdullah bin Abdulaziz University Hospital, Princess Nourah bint Abdulrahman University, Riyadh 11564, Saudi Arabia

**Keywords:** primary ovarian insufficiency, adolescents, hormone replacement therapy, fertility counselling, bone mineral density, psychological distress, reproductive endocrinology, quality of life, diagnostic delay, multidisciplinary care

## Abstract

Primary ovarian insufficiency (POI) in adolescents and young women is a rare but serious endocrine disorder with far-reaching reproductive, metabolic, and psychological implications. This study aimed to evaluate diagnostic timelines, treatment patterns, and psychosocial outcomes among affected individuals in a secondary care setting in Saudi Arabia. A retrospective observational analysis was conducted on 96 patients aged 13–39 years diagnosed with POI between 2018 and 2024. Data were extracted from electronic medical records and assessed using validated clinical and psychological tools, including the MENQOL and HADS. The mean age at diagnosis was 22.9 years, with one-third of patients experiencing diagnostic delays exceeding 18 months. Hormone replacement therapy was initiated in 69.8% of cases, while fertility counselling and bone mineral density screening were provided to 61.5% and 74.0% of patients, respectively. Over 60% exhibited clinically significant symptoms of anxiety or depression. Multivariate analysis revealed that delayed diagnosis, absence of hormone therapy, and lack of fertility counselling significantly increased the risk of psychological distress. These findings underscore the importance of timely diagnosis, multidisciplinary intervention, and integrated mental health support in the management of POI. Comprehensive, culturally responsive care models are essential to improving quality of life and long-term outcomes in this vulnerable population.

## 1. Introduction

Primary ovarian insufficiency (POI), formerly termed premature ovarian failure, represents a significant and complex reproductive endocrine disorder characterized by impaired ovarian function in women under the age of 40, marked by hypoestrogenism, elevated gonadotropins, and menstrual irregularity or amenorrhea [1,2]. Although traditionally associated with menopause, POI diverges in etiology and clinical implications, particularly when it manifests in adolescents and young women [3]. This early disruption in ovarian function has profound physiological, psychological, and developmental implications, making its timely diagnosis and evidence-based management a critical concern in reproductive medicine [4].

The incidence of POI is estimated to affect approximately 1% of women under the age of 40, 0.1% of those under 30, and as low as 0.01% of those under 20 [5]. Despite its relatively low prevalence in younger age groups, the consequences of POI in adolescents and young women are substantial. This condition not only impairs fertility but also affects bone health, cardiovascular function, cognitive development, and psychological wellbeing [6]. Given the pivotal role of estrogen in the development of secondary sexual characteristics and neuroendocrine maturation during adolescence, the onset of POI during this critical period of development can significantly hinder physical and emotional health outcomes [7]. Given that POI can present in mid- to late-adolescence and early adulthood, our cohort intentionally includes adolescents (13–17) and young women (18–39) to permit age-stratified comparisons and to isolate management considerations unique to minors [8].

The etiology of POI is multifactorial and includes genetic, autoimmune, iatrogenic, and idiopathic causes. Genetic contributions, particularly X chromosome abnormalities such as Turner syndrome and Fragile X premutations, are among the most common identifiable causes in adolescents [9,10]. Autoimmune oophoritis and iatrogenic insults, such as chemotherapy or pelvic radiation, are also well-documented contributors [11]. However, in many cases, no specific etiology is identified, highlighting the complex interplay of genetic and environmental factors that underlie ovarian dysfunction [12]. A thorough understanding of these etiologies is essential not only for diagnosis and treatment but also for counselling on long-term health implications and fertility options.

The diagnostic criteria for POI in adolescents and young women must be approached with particular sensitivity and caution. The natural variability of menstrual cycles in adolescence can obscure early symptoms, often leading to delayed diagnosis and treatment initiation [13]. According to current guidelines, the diagnosis of POI requires at least four months of amenorrhea or menstrual irregularity and two elevated serum follicle-stimulating hormone (FSH) measurements in the menopausal range taken at least one month apart [14]. Additionally, assessment of estradiol levels, anti-Müllerian hormone (AMH), and ovarian imaging may contribute to the diagnostic workup [15]. Importantly, such evaluations must be framed within the context of the patient’s developmental stage, psychosocial readiness, and family involvement in healthcare decision-making.

The management of POI in adolescents and young women necessitates a multidisciplinary and individualized approach that addresses both medical and psychosocial needs. Hormone replacement therapy (HRT) remains the cornerstone of treatment, aiming to restore estrogen levels to support normal pubertal progression, maintain bone mineral density, and alleviate vasomotor symptoms [16]. Cyclic estrogen-progestin regimens are preferred to mimic physiological patterns and protect against endometrial hyperplasia [17]. However, the initiation and monitoring of HRT in this age group require consideration of developmental, behavioural, and adherence factors that may differ from older women with postmenopausal symptoms [18].

Beyond hormonal therapy, management should include early, realistic fertility counselling. For adolescents and young women who already meet diagnostic criteria for POI, conventional mature oocyte cryopreservation is often not feasible due to markedly diminished follicular reserve. Instead, counselling should focus on the low but non-zero chance of intermittent ovarian activity and spontaneous conception, options for future family building (e.g., oocyte donation or adoption), and—when relevant—fertility preservation prior to gonadotoxic therapy in at-risk patients, as recommended by international guidelines [19,20]. Although spontaneous pregnancy is rare, it is not impossible, occurring in approximately 5–10% of affected women, and should be discussed realistically during counselling [21]. Moreover, patients must be supported with information regarding options for adoption, emphasizing autonomy and psychological wellbeing in decision-making.

The psychosocial impact of POI on adolescents and young women cannot be overstated. The diagnosis often results in emotional distress, depression, anxiety, and a diminished sense of femininity and identity, particularly in cultures where fertility is closely linked to self-worth and societal roles [22]. Comprehensive care models should incorporate routine mental-health screening (e.g., HADS), brief cognitive-behavioural psychoeducation, and moderated, confidential, online peer communities for infertility/POI more broadly, which have demonstrated acceptability and symptom benefits [23]. When condition-specific peers are scarce, condition-agnostic adolescent chronic-illness groups and one-to-one counselling can provide comparable social support without requiring public disclosure [24,25]. In particular, healthcare providers must cultivate trust and empathy to empower patients in navigating this life-altering condition.

Despite advances in the understanding and treatment of POI, evidence-based guidelines specific to adolescents and young women remain limited. Most current recommendations are extrapolated from adult data, which may not fully address the unique developmental, endocrinologic, and psychosocial needs of this population [26]. There is a critical need for age-specific protocols that integrate current scientific evidence with patient-centred care principles. Furthermore, early identification through improved clinical awareness and enhanced diagnostic tools could help mitigate long-term consequences and improve quality of life outcomes for affected individuals.

### 1.1. Aim of the Study

**Aim**: To describe diagnostic timelines, management patterns, bone and psychological outcomes in adolescents and young women with POI in a secondary-care setting, and to examine predictors of psychological distress.

**Secondary Aim**: To compare adolescents (<18 years) with those ≥18 years to assess whether care-process and outcome differences justify age-tailored care pathways.

### 1.2. Research Questions

1. What are the most effective evidence-based diagnostic criteria and protocols for identifying POI in adolescents and young women?
2. What are the current best practices in the medical and psychosocial management of POI in this population?

## 2. Materials and Methods

### 2.1. Study Design

This was a retrospective, observational cohort study of consecutive patients with a confirmed diagnosis of primary ovarian insufficiency (POI) managed at a single tertiary/secondary care centre. We followed STROBE recommendations for reporting observational studies. Given the rarity of POI in adolescence, a retrospective design was chosen to maximize sample size and reflect real-world practice.

### 2.2. Setting

The study was conducted at KAAUH, a secondary/tertiary care hospital with integrated electronic medical records (EMR). The centre provides gynecology/endocrinology services, including diagnostic evaluation, hormone replacement therapy (HRT), bone health assessment, fertility counselling, and psychological screening.

### 2.3. Sample and Sampling

We included all females aged 13–39 years who met diagnostic criteria for POI and had at least one documented clinic visit during the study period. Exclusion criteria were iatrogenic/surgical menopause without POI, incomplete records for the primary outcomes, or concurrent conditions that preclude HRT. To address Reviewer 1’s concern regarding age specificity, we prespecified an age-stratified analysis: adolescents (<18 years) and young adults (≥18 years).

Baseline variables abstracted from the EMR included: age at diagnosis and at index visit, diagnostic delay (months from first amenorrhea/oligo-amenorrhea or suggestive symptom to confirmed diagnosis), marital status, parity, prior live birth (yes/no), prior fertility treatment (yes/no), and (when documented) completed family size/reproductive plan. Clinical data included BMI, relevant laboratory results, bone mineral density (BMD) testing, and documentation of fertility counselling referral/attendance.

### 2.4. Measures and Data Sources

Data were abstracted using a standardized Clinical Management Record Form (CMRF) developed by the research team and piloted on a random subset of charts before full extraction. Psychological and quality-of-life outcomes were captured using validated Arabic versions of the Hospital Anxiety and Depression Scale (HADS) and the Menopause-Specific Quality of Life Questionnaire (MENQOL). HADS subscales range 0–21; consistent with prior literature, scores ≥ 8 on either subscale were considered indicative of clinically relevant symptoms. MENQOL domain and total scores were computed per instrument guidance to index vasomotor, psychosocial, physical, and sexual symptom burden in hypoestrogenic states.

#### 2.4.1. Hormone Replacement Therapy (HRT) Abstraction

For participants prescribed HRT, we extracted estrogen formulation (e.g., 17β-estradiol, ethinyl estradiol, estradiol valerate), dose, route (oral, transdermal, other), progestogen type and schedule (continuous vs. cyclic), combined oral contraceptive use (yes/no), time from diagnosis to HRT initiation (months), and—when documented—reasons for non-initiation or discontinuation (e.g., side effects, cost, preference).

#### 2.4.2. Qualitative Free-Text Abstraction

To complement quantitative measures, EMR free-text clinical notes were reviewed for patient-reported concerns documented during consultations (e.g., fertility/identity loss, stigma/disclosure, medication beliefs/adherence). Using a rapid content approach, two reviewers independently screened notes for relevant statements, coded recurrent descriptive themes, and resolved differences by discussion. Findings are presented descriptively to contextualize the quantitative results; given the retrospective nature and documentation bias, no inferential qualitative analyses were attempted.

### 2.5. Operational Definitions

- Age group: adolescents < 18 vs. young adults ≥ 18 (pre-specified).
- Diagnostic delay: months from first suggestive symptom to confirmed POI diagnosis.
- HRT initiation within 3 months: initiation ≤ 3 months after diagnosis (yes/no).
- Fertility counselling: documented referral and/or attendance (yes/no).
- BMD screening: any DXA scan within the study period (yes/no).
- Clinically elevated anxiety/depression: HADS-A or HADS-D ≥ 8.

Where “completed family size/reproductive plan” was not explicitly documented, this is reported as missing and discussed in the Limitations.

### 2.6. Statistical Analysis

Continuous variables were summarized as mean ± SD or median (IQR) based on distribution (assessed by Shapiro–Wilk and visual inspection). Categorical variables were summarized as counts (percentages).

Age-stratified analysis: We compared adolescents (<18) vs. adults (≥18) for diagnostic delay, HRT initiation within 3 months, fertility counselling, BMD screening, MENQOL total/domains, and HADS-A/HADS-D. Between-group differences used *t*-tests or Mann–Whitney U tests for continuous variables and χ^2^ or Fisher’s exact tests for categorical variables. We report effect sizes (Cohen’s *d* for continuous outcomes; odds ratios [OR] with 95% CIs for categorical outcomes).

Multivariable modelling: To explore correlates of psychological distress, we fitted logistic regression models with HADS-A ≥ 8 and HADS-D ≥ 8 as outcomes. Prespecified predictors included age group, diagnostic delay (categorized), HRT initiation within 3 months, fertility counselling, and parity; additional clinical covariates were added based on clinical relevance and bivariate screening. Model diagnostics included multicollinearity assessment and Hosmer-Lemeshow goodness-of-fit.

Comparative context (no control group): In the absence of an internal control cohort, we contextualized HADS and MENQOL findings against published population norms and prior POI cohorts (descriptive comparison only, acknowledging differences in age and setting).

Missing data: Analyses were conducted on a complete-case basis for each outcome; the extent of missingness is reported per variable. Two-sided *p* < 0.05 denoted statistical significance. Analyses were performed using IBM SPSS 27 (or equivalent) and R package version 0.3.1 where appropriate.

### 2.7. Ethical Considerations

Ethical approval for the study was granted by the Institutional Review Board (IRB) of King Abdullah bin Abdulaziz University Hospital under IRB Log Number: 25-0072, with the category of expedited review due to minimal risk to participants. All data were anonymized to protect patient identity. As this was a retrospective chart review. However, strict data security protocols were maintained, and access was limited to authorized research team members only. Researchers involved in the study were required to complete institutional training in research ethics and confidentiality prior to data access.

## 3. Results

Table 1 presents the demographic and baseline clinical characteristics of the 96 participants with POI, stratified by age group (<18 years vs. ≥18 years). Adolescents comprised 14.6% of the cohort (n = 14). As expected, there were statistically significant differences in both current age and age at diagnosis between groups, with adolescents diagnosed at a mean of 16.5 years compared with 24.0 years in adults (*p* < 0.001 for both), reflecting the study’s age-group definition.

Median symptom duration prior to diagnosis was longer in adolescents (20 months) than in adults (15 months), a difference that reached statistical significance (*p* = 0.041, r = 0.21). This finding is consistent with a potential diagnostic delay in younger patients, possibly related to challenges distinguishing pathological amenorrhoea from normal pubertal variation.

BMI and baseline FSH values did not differ significantly between groups (*p* > 0.3 for both), suggesting comparable anthropometric and hormonal profiles at presentation. The overall median FSH level in the cohort was elevated, as expected in established POI, with no evidence of a clinically meaningful age-group difference (Cohen’s *d* = 0.15).

Table 2 shows Quartile analysis revealed that one-quarter of patients had profoundly elevated FSH (>59 × 8 IU·L^−1^) that reflecting intermittent follicular escape. Anti-Müllerian hormone (AMH) values clustered near the assay’s lower technical limit yet retained variability, suggesting residual follicular activity in a minority. Luteinising hormone (LH) mirrored FSH dispersion, reinforcing the centrality of the hypothalamic-pituitary-gonadal axis in pathophysiology.

Nearly seven-in-ten patients commenced hormone-replacement therapy (HRT), with a gratifyingly short median initiation lag of 1 × 3 mo. Nevertheless, roughly three-in-ten remained untreated, often citing fertility hopes or parental hesitation. Fertility counselling, delivered to 59 patients, lagged behind diagnosis by almost four months—a gap that can erode ovarian reserve options. Bone mineral density (BMD) screening coverage was high (71 patients), but the median scheduling at 4 × 4 mo risks early bone mass loss in the most hypo-estrogenic group (Table 3).

As shown in Table 4 Among those screened, nearly one-third already demonstrated low-bone-mass states (osteopenia or osteoporosis), underscoring the silent skeletal cost of untreated hypo-estrogenism. Mean serum 25-hydroxy-vitamin-D (27 × 6 ± 6 × 7 ng·mL^−1^) hovered below the recommended target, compounding fracture risk and suggesting the need for routine supplementation protocols.

Domain-specific MENQOL scoring exposed a disproportionately high physical burden (16 × 3 ± 4 × 1) driven by fatigue, musculo-skeletal pain, and sleep fragmentation, followed closely by psychosocial distress (14 × 1 ± 3 × 6). Vasomotor complaints, while expectedly present, ranked third in severity, revealing that counselling focused solely on ‘hot flushes’ risks trivializing the broader symptom complex. Sexual-function scores were lowest, yet qualitative chart notes revealed avoidance rather than resolution, hinting at under-reporting in unmarried adolescents (Table 5).

As shown in Table 6a,b, Clinical thresholds on the Hospital Anxiety and Depression Scale (HADS) were exceeded by 60 patients for anxiety and 56 for depression. Severity stratification (Table 6a) shows a worrisome rightward shift toward moderate–severe categories. Multivariable modelling (Table 6b) revealed that delayed diagnosis, absence of HRT, and lack of fertility counselling each independently doubled the odds of psychological distress—a triad readily amenable to systems-level quality-improvement interventions.

A targeted review of free-text clinical notes yielded additional contextual information regarding the lived experiences and care-related concerns of participants. Four recurring descriptive themes were identified: (1) fertility and identity loss, including expressions of grief and uncertainty regarding future family building; (2) stigma and disclosure concerns, particularly fear of negative reactions if the diagnosis were shared with family or peers; (3) medication beliefs and adherence, including apprehension about side effects of HRT and issues related to cost and availability; and (4) care navigation needs, such as a desire for clearer counselling and more coordinated follow-up across specialties. Representative excerpts are summarized in Table 7.

## 4. Discussion

This single-centre retrospective cohort of 96 adolescents and young women with primary ovarian insufficiency (POI) adds practice-relevant data from a Middle Eastern setting by integrating clinical management patterns (HRT, bone health, fertility counselling) with symptom and psychological measures (MENQOL, HADS) and targeted review of clinical notes. Three findings are central. First, diagnostic delay remains common and was longer among adolescents. Second, HRT uptake approached 70% with early initiation in most cases, yet one-third of those screened already had low bone mass, underscoring the cost of delayed estrogen replacement. Third, clinically meaningful anxiety and depressive symptoms were frequent; in adjusted analyses, longer delay and absence of timely HRT were the most consistent correlates of psychological distress. Together, these observations support age-tailored implementation of established international guidance rather than proposing new disease-specific guidelines for adolescents.

### 4.1. Diagnostic Timeliness and Age-Specific Considerations

Adolescents showed a statistically significant longer median interval from symptom onset to POI diagnosis compared with adults. This is consistent with literature noting that menstrual irregularity in mid-to-late adolescence is often normalized or attributed to functional hypothalamic causes, delaying definitive endocrine evaluation [27,28]. Our data suggest that targeted prompts (e.g., “amenorrhoea/oligo-amenorrhoea > 3 months with vasomotor symptoms → check FSH/LH twice, ≥4 weeks apart”) and expedited referral pathways for minors could reduce time to diagnosis. While hormonal and anthropometric profiles did not differ meaningfully by age, earlier recognition in adolescents has outsized implications for pubertal progression, bone accrual, and psychosocial adjustment [29,30].

### 4.2. HRT Patterns, Bone Health, and Alignment with Guidance

Approximately 70% initiated HRT, typically within two to three months of diagnosis, and regimens largely reflected physiologic estradiol (transdermal 17β-E2 or oral estradiol valerate) with appropriate progestogen protection, in line with current ESHRE and IMS recommendations [31,32]. Nonetheless, ~32% of those with DXA had low bone mass at baseline, indicating that bone loss often precedes therapy. This supports clear operational targets: (i) earlier initiation of estradiol in confirmed POI (or induction where puberty is incomplete), (ii) systematic BMD screening at diagnosis with interval follow-up, and (iii) adherence support. Our findings do not justify altering ESHRE/IMS guidelines but highlight implementation gaps, particularly for minors, such as prompt DXA ordering, default transdermal estradiol for adolescents when feasible, and proactive scheduling to minimize lapses in access [33].

### 4.3. Psychological Burden and Supportive Care

Over one-third screened positive for anxiety and nearly one-third for depression on HADS. This prevalence is higher than that reported in age-matched community populations [34,35] and aligns with prior POI studies linking early estrogen deficiency to poorer mental health outcomes [36]. In multivariable models, diagnostic delay ≥12 months and no timely HRT were associated with higher odds of anxiety/depression, suggesting that both disease trajectory and care processes shape mental health. Routine screening with HADS (threshold ≥ 8), followed by stepped care—brief psychoeducation and cognitive-behavioural strategies for mild/moderate distress, referral for persistent symptoms—is warranted [37,38].

Our qualitative review of clinical notes revealed recurring themes—fertility/identity loss, stigma and disclosure concerns, medication beliefs/adherence, and care navigation needs—that complement the quantitative findings and point to service improvements. Such concerns are echoed in qualitative research among women with POI in diverse settings, where fertility loss, identity shifts, and healthcare system navigation difficulties are recurrent [39].

### 4.4. Fertility Counselling: Realistic Options Post-Diagnosis

We have revised our framing to avoid implying that oocyte cryopreservation is feasible after a POI diagnosis. For those already meeting diagnostic criteria, diminished follicular reserve typically precludes mature oocyte banking [40]. Counselling should therefore emphasize: (i) the low but non-zero probability of intermittent ovarian activity and spontaneous conception [41]; (ii) realistic family-building options (oocyte donation, embryo donation, adoption); and (iii) fertility preservation prior to gonadotoxic therapy for at-risk girls and young women [42]. This approach aligns with international guidance while setting accurate expectations for adolescents and young adults.

### 4.5. Cultural Considerations and Generalisability

In our setting, cultural norms surrounding fertility, marriageability, and disclosure can influence health-seeking behaviour, acceptance of HRT, and willingness to pursue assisted reproduction [43]. While these influences are heterogeneous and individually mediated, they should be recognized in service planning. Comparative data suggest that in more individualized cultures, reproductive decision-making is less constrained by such factors [44], underscoring the need for culturally sensitive, patient-centred approaches. Our findings are from a single tertiary centre and may not generalize to all healthcare systems or cultural contexts.

### 4.6. Strengths and Limitations

This study offers several notable strengths. First, it addresses a rare but clinically significant condition in an understudied population, integrating clinical, psychosocial, and cultural dimensions of care for adolescents and young women with POI. Second, the cohort size of 96 patients is relatively large for a single-centre study in this demographic, enhancing the robustness of subgroup analyses. Third, the use of validated, culturally adapted tools—the Arabic version of the MENQOL and the locally validated HADS—strengthens the reliability and cultural relevance of patient-reported outcomes. Fourth, the integration of qualitative insights from clinical notes adds contextual depth, capturing lived experiences and barriers to care that are often overlooked in purely quantitative analyses.

Nevertheless, several limitations warrant consideration. The retrospective design and reliance on electronic medical records may introduce documentation bias, particularly for qualitative themes and certain baseline variables such as reproductive intentions or completed family size. The absence of a control group limits the ability to contextualize findings against normative psychological and quality-of-life benchmarks. Conducting the study at a single tertiary centre in Saudi Arabia may restrict generalisability to other healthcare settings and cultural contexts. Longitudinal follow-up data on bone health, ovarian function, and psychosocial status were not available, precluding assessment of the long-term impact of interventions. Finally, while the adolescent subgroup was analyzed separately, the relatively small number of patients < 18 years reduces statistical power for detecting between-group differences.

### 4.7. Implications and Future Directions

The findings underscore the need for timely diagnosis, early initiation of physiologic estrogen replacement, and comprehensive fertility counselling tailored to the developmental stage of the patient. For adolescents, reducing diagnostic delay through heightened clinical awareness and streamlined referral pathways is critical to preserving bone mass, supporting pubertal development, and mitigating psychological distress. The high prevalence of anxiety and depression highlights the importance of embedding routine mental health screening and stepped-care interventions into POI management protocols.

Cultural influences on disclosure, HRT acceptance, and reproductive decision-making require locally tailored strategies, such as culturally sensitive counselling scripts, confidential patient education platforms, and discreet peer or online support groups. Implementation research should evaluate how best to adapt existing ESHRE and IMS guidelines for adolescent service delivery without altering their evidence-based core.

Future studies should adopt prospective, multicentre designs with matched control groups to contextualize patient-reported outcomes and track long-term trajectories in bone health, ovarian function, and mental wellbeing. Embedding mixed-methods approaches will further illuminate the interplay between clinical interventions, sociocultural factors, and patient priorities. Such work will be essential to refining care models that are both evidence-based and contextually relevant, ensuring that adolescents and young women with POI receive comprehensive, patient-centred support.

## 5. Conclusions

In conclusion, this study provides a detailed, evidence-based examination of the diagnostic timelines, management patterns, and psychosocial burden associated with primary ovarian insufficiency in adolescents and young women in a secondary-care setting in Saudi Arabia. Despite relatively high rates of clinical intervention, delays in diagnosis and underutilization of fertility counselling remain significant challenges. Moreover, the high prevalence of psychological distress and compromised quality of life among affected individuals underscores the multidimensional impact of POI during formative developmental years. Prompt recognition, early hormone replacement therapy, integrated fertility planning, and mental health support must be prioritized in the management of POI. Future research should focus on longitudinal outcomes, culturally tailored psychosocial interventions, and the development of region-specific clinical practice guidelines to enhance the care and wellbeing of young women living with this complex condition.

## Figures and Tables

**Table 1:** Demographic and baseline clinical characteristics.

Variable	Total (N = 96)	Adolescents < 18 (n = 14)	Adults ≥ 18 (n = 82)	Test (t/U/χ^2^)	*p*-Value	Effect Size (d/r/OR)
Current age (years), mean ± SD	24.8 ± 5.4	17.2 ± 0.8	26.0 ± 4.0	t = −10.62	<0.001	d = 2.56
Age at diagnosis (years), mean ± SD	22.9 ± 3.1	16.5 ± 0.7	24.0 ± 2.1	t = −13.87	<0.001	d = 3.59
BMI (kg/m^2^), mean ± SD	23.7 ± 3.9	22.9 ± 3.5	23.8 ± 4.0	t = −0.85	0.398	d = 0.23
FSH (IU/L), mean ± SD	52.8 ± 9.6	54.1 ± 8.9	52.6 ± 9.7	t = 0.53	0.597	d = 0.15
Symptom duration (months), median (IQR)	16 (9–24)	20 (12–26)	15 (9–24)	U = 423.5	0.041	r = 0.21

**Table 2:** Endocrine Profile.

Parameter	Q1	Median	Q3	Range/Mean ± SD
FSH (IU·L^−1^)	44 × 7	52 × 4	59 × 8	33 × 4–68 × 9
LH (IU·L^−1^)	-	-	-	28 × 9 ± 6 × 4
AMH (ng·mL^−1^)	-	-	-	0 × 13 ± 0 × 04

**Table 3:** Uptake, Timing, and Breadth of Core Interventions.

Intervention	n (%)	Median Start–Dx Lag (mo)|IQR
HRT initiated	67 (69 × 8%)	1 × 3 | 0 × 9–2 × 4
Fertility counselling	59 (61 × 5%)	3 × 7 | 1 × 2–6 × 9
BMD screening	71 (74 × 0%)	4 × 4 | 2 × 1–7 × 2

**Table 4:** Skeletal Health Snapshot.

BMD Category	n (%)
Normal (T > −1)	46 (64 × 8%)
Osteopenia (−2 × 5 < T ≤ −1)	19 (26 × 8%)
Osteoporosis (T ≤ −2 × 5)	6 (8 × 4%)
Vitamin D (ng·mL^−1^)	27 × 6 ± 6 × 7

**Table 5:** Quality-of-Life Burden (MENQOL).

MENQOL Domain	Mean ± SD	Median (IQR)
Vasomotor	11 × 8 ± 3 × 3	12 (9–14)
Psychosocial	14 × 1 ± 3 × 6	14 (12–17)
Physical	16 × 3 ± 4 × 1	16 (13–19)
Sexual	5 × 1 ± 2 × 5	5 (3–7)
Total Score	47 × 3 ± 8 × 9	46 (40–53)

**Table 6:** (a): Severity Distribution of HADS Scores. (b): Multivariate Logistic Regression Predicting Psychological Distress (HADS ≥ 8).

(**a**)		
**Severity**	**Anxiety n (%)**	**Depression n (%)**
Normal (0–7)	36 (37 × 5%)	40 (41 × 7%)
Mild (8–10)	22 (22 × 9%)	18 (18 × 8%)
Moderate (11–14)	26 (27 × 1%)	24 (25 × 0%)
Severe (≥15)	12 (12 × 5%)	14 (14 × 6%)
(**b**)		
**Predictor**	**Odds Ratio (95% CI)**	***p*** **-Value**
Symptom-to-diagnosis interval > 18 mo	2 × 3 (1 × 2–4 × 2)	0 × 006
No HRT	1 × 8 (1 × 1–3 × 1)	0 × 021
No fertility counselling	2 × 2 (1 × 2–3 × 8)	0 × 013
Age at diagnosis	1 × 0 (0 × 9–1 × 1)	0 × 327

**Table 7:** Illustrative Excerpts.

Theme	Illustrative Excerpts	Frequency of Occurrence *
Fertility and identity loss	“I feel less of a woman knowing I may never have my own children.”; “I’m worried about my future as a wife if I cannot get pregnant.”	23 (24.0%)
Stigma and disclosure concerns	“I have not told my friends or relatives; they will not understand.”; “I’m afraid my fiancé will call off the wedding if he knows.”	15 (15.6%)
Medication beliefs and adherence	“I stopped the pills because they made me feel bloated.”; “The pharmacy didn’t have my patch in stock for two months.”	19 (19.8%)
Care navigation needs	“I don’t know which doctor I should follow up with for my bone scans.”; “I wish someone explained the steps more clearly.”	12 (12.5%)

* Frequency refers to the number of participants whose clinical notes contained at least one statement matching the theme. Percentages are calculated from the total cohort (N = 96).

## Data Availability

The data presented in this study are available on reasonable request from the corresponding author. The data are not publicly available due to institutional privacy policies and ethical restrictions.

## References

[1] Torrealday S., Kodaman P., Pal L. (2017). Premature Ovarian Insufficiency—An Update on Recent Advances in Understanding and Management. F1000Research.

[2] Chon S.J., Umair Z., Yoon M.-S. (2021). Premature Ovarian Insufficiency: Past, Present, and Future. Front. Cell Dev. Biol..

[3] Armeni E. (2019). Investigating Menopause in Adolescent Girls. Case Rep. Women’s Health.

[4] Witchel S.F., Oberfield S.E., Peña A.S. (2019). Polycystic Ovary Syndrome: Pathophysiology, Presentation, and Treatment with Emphasis on Adolescent Girls. J. Endocr. Soc..

[5] Dragojević-Dikić S., Marisavljević D., Mitrović A., Dikić S., Jovanović T., Janković-Ražnatović S. (2010). An Immunological Insight into Premature Ovarian Failure (POF). Autoimmun. Rev..

[6] Federici S., Rossetti R., Moleri S., Munari E.V., Frixou M., Bonomi M., Persani L. (2024). Primary Ovarian Insufficiency: Update on Clinical and Genetic Findings. Front. Endocrinol..

[7] Carnesi E., Castellano S., Albani E., Busnelli A., Smeraldi A., Bulbul O., Morenghi E., Immediata V., Levi-Setti P.E. (2025). Diminished Ovarian Reserve Is Associated to Euploidy Rate: A Single Center Study. Front. Endocrinol..

[8] Kanner L., Hakim J.C.E., Davis Kankanamge C., Patel V., Yu V., Podany E., Gomez-Lobo V. (2018). Noncytotoxic-Related Primary Ovarian Insufficiency in Adolescents: Multicenter Case Series and Review. J. Pediatr. Adolesc. Gynecol..

[9] Tucker E.J., Grover S.R., Bachelot A., Touraine P., Sinclair A.H. (2016). Premature Ovarian Insufficiency: New Perspectives on Genetic Cause and Phenotypic Spectrum. Endocr. Rev..

[10] Nie L., Wang X., Wang S., Hong Z., Wang M. (2024). Genetic Insights into the Complexity of Premature Ovarian Insufficiency. Reprod. Biol. Endocrinol..

[11] Morris K. (2015). AL Pelvic Radiation Therapy: Between Delight and Disaster. World J. Gastrointest. Surg..

[12] Khan M.J., Ullah A., Basit S. (2019). Genetic Basis of Polycystic Ovary Syndrome (PCOS): Current Perspectives. Appl. Clin. Genet..

[13] Committee Opinion No. 605. (2014). Primary Ovarian Insufficiency in Adolescents and Young Women. Obstet. Gynecol..

[14] Davis J.B., Segars J.H. (2009). Menstruation and Menstrual Disorders: Anovulation. Glob. Libr. Women’s Med..

[15] Moolhuijsen L.M.E., Visser J.A. (2020). Anti-Müllerian Hormone and Ovarian Reserve: Update on Assessing Ovarian Function. J. Clin. Endocrinol. Metab..

[16] Sullivan S.D., Sarrel P.M., Nelson L.M. (2016). Hormone Replacement Therapy in Young Women with Primary Ovarian Insufficiency and Early Menopause. Fertil. Steril..

[17] Ettinger B., Selby J., Citron J.T., Vangessel A., Ettinger V.M., Hendrickson M.R. (1994). Cyclic Hormone Replacement Therapy Using Quarterly Progestin. Obstet. Gynecol..

[18] Khan S.J., Kapoor E., Faubion S.S., Kling J.M. (2023). Vasomotor Symptoms During Menopause: A Practical Guide on Current Treatments and Future Perspectives. Int. J. Women’s Health.

[19] Cousineau T.M., Green T.C., Corsini E., Seibring A., Showstack M.T., Applegarth L., Davidson M., Perloe M. (2008). Online Psychoeducational Support for Infertile Women: A Randomized Controlled Trial. Hum. Reprod..

[20] Yang E.H., Strohl H.B., Su H.I. (2024). Fertility Preservation before and after Cancer Treatment in Children, Adolescents, and Young Adults. Cancer.

[21] Simpson J.L., Rechitsky S., Kuliev A. (2019). Before the Beginning: The Genetic Risk of a Couple Aiming to Conceive. Fertil. Steril..

[22] Hasanpoor-Azghdy S.B., Simbar M., Vedadhir A. (2014). The Emotional-Psychological Consequences of Infertility among Infertile Women Seeking Treatment: Results of a Qualitative Study. Iran. J. Reprod. Med..

[23] Koochaksaraei F.Y., Simbar M., Khoshnoodifar M., Faramarzi M., Nasiri M. (2023). Interventions Promoting Mental Health Dimensions in Infertile Women: A Systematic Review. BMC Psychol..

[24] Massarotti C., Gentile G., Ferreccio C., Scaruffi P., Remorgida V., Anserini P. (2019). Impact of Infertility and Infertility Treatments on Quality of Life and Levels of Anxiety and Depression in Women Undergoing in Vitro Fertilization. Gynecol. Endocrinol..

[25] Wiedermann C.J., Barbieri V., Plagg B., Marino P., Piccoliori G., Engl A. (2023). Fortifying the Foundations: A Comprehensive Approach to Enhancing Mental Health Support in Educational Policies Amidst Crises. Healthcare.

[26] Panay N., Anderson R.A., Bennie A., Cedars M., Davies M., Ee C., Gravholt C.H., Kalantaridou S., Kallen A., Kim K.Q. (2024). Evidence-Based Guideline: Premature Ovarian Insufficiency † ‡. Climacteric.

[27] Abacı A. (2024). Delayed Puberty and Management of Treatment. J. Clin. Res. Pediatr. Endocrinol..

[28] Rajiwade S.R., Sagili H., Soundravally R., Subitha L. (2018). Endocrine Abnormalities in Adolescents with Menstrual Disorders. J. Obstet. Gynecol. India.

[29] Sophie Gibson M.E., Fleming N., Zuijdwijk C., Dumont T. (2020). Where Have the Periods Gone? The Evaluation and Management of Functional Hypothalamic Amenorrhea. J. Clin. Res. Pediatr. Endocrinol..

[30] Newbery G., Neelakantan M., Cabral M.D., Omar H. (2019). Amenorrhea in Adolescents: A Narrative Review. Pediatr. Med..

[31] Memi E., Pavli P., Papagianni M., Vrachnis N., Mastorakos G. (2024). Diagnostic and Therapeutic Use of Oral Micronized Progesterone in Endocrinology. Rev. Endocr. Metab. Disord..

[32] Casanova G., Spritzer P.M. (2012). Effects of Micronized Progesterone Added to Non-Oral Estradiol on Lipids and Cardiovascular Risk Factors in Early Postmenopause: A Clinical Trial. Lipids Health Dis..

[33] Malhi G.S., Bell E., Bassett D., Boyce P., Bryant R., Hazell P., Hopwood M., Lyndon B., Mulder R., Porter R. (2021). The 2020 Royal Australian and New Zealand College of Psychiatrists Clinical Practice Guidelines for Mood Disorders. Aust. N. Z. J. Psychiatry.

[34] Sivertsen H.E., Helvik A.-S., Gjøra L., Haugan G. (2023). Psychometric Validation of the Hospital Anxiety and Depression Scale (HADS) in Community-Dwelling Older Adults. BMC Psychiatry.

[35] Nour M.O., Alharbi K.K., Hafiz T.A., Alshehri A.M., Alyamani L.S., Alharbi T.H., Alzahrani R.S., Almalki E.F., Althagafi A.A., Kattan E.T. (2023). Prevalence of Depression and Associated Factors among Adults in Saudi Arabia: Systematic Review and Meta-Analysis (2000–2022). Depress. Anxiety.

[36] Yuan S., Gong Y., Zhang Y., Cao W., Wei L., Sun T., Sun J., Wang L., Zhang Q., Wang Q. (2025). Brain Structural Alterations in Young Women with Premature Ovarian Insufficiency: Implications for Dementia Risk. Alzheimer’s Dement..

[37] Collister D., Rodrigues J.C., Mazzetti A., Salisbury K., Morosin L., Rabbat C., Brimble K.S., Walsh M. (2019). Single Questions for the Screening of Anxiety and Depression in Hemodialysis. Can. J. Kidney Health Dis..

[38] Nordin K., Berglund G., Glimelius B., Sjödén P.-O. (2001). Predicting Anxiety and Depression among Cancer Patients: A Clinical Model. Eur. J. Cancer.

[39] Golezar S., Keshavarz Z., Ramezani Tehrani F., Ebadi A. (2020). An Exploration of Factors Affecting the Quality of Life of Women with Primary Ovarian Insufficiency: A Qualitative Study. BMC Women’s Health.

[40] Man L., Lustgarten Guahmich N., Vyas N., Tsai S., Arazi L., Lilienthal D., Schattman G., Rosenwaks Z., James D. (2022). Ovarian Reserve Disorders, Can We Prevent Them? A Review. Int. J. Mol. Sci..

[41] Moustaki M., Kontogeorgi A., Tsangkalova G., Tzoupis H., Makrigiannakis A., Vryonidou A., Kalantaridou S.N. (2023). Biological Therapies for Premature Ovarian Insufficiency: What Is the Evidence?. Front. Reprod. Health.

[42] Santulli P., Blockeel C., Bourdon M., Coticchio G., Campbell A., De Vos M., Macklon K.T., Pinborg A., Garcia-Velasco J.A. (2023). Fertility Preservation in Women with Benign Gynaecological Conditions. Hum. Reprod. Open.

[43] Ouahid H., Sebbani M., Cherkaoui M., Amine M., Adarmouch L. (2025). The Influence of Gender Norms on Women’s Sexual and Reproductive Health Outcomes: A Systematic Review. BMC Women’s Health.

[44] Miskeen E., Korkoman S., Alhassoun N.K., Aljuhani R.F., Alqahtani R.A.H., Alwabari S.S., Alamri M.S., Alshahrani A.M., Alhalafi A.H., Almalki S. (2025). Factors Influencing Family Planning Decisions in Saudi Arabia. BMC Women’s Health.

